# Contingency, repeatability, and predictability in the evolution of a prokaryotic pangenome

**DOI:** 10.1073/pnas.2304934120

**Published:** 2023-12-26

**Authors:** Alan Beavan, Maria Rosa Domingo-Sananes, James O. McInerney

**Affiliations:** ^a^School of Life Sciences, The University of Nottingham, Nottingham NG7 2UH, United Kingdom; ^b^School of Science and Technology, Nottingham Trent University, Nottingham NG1 4FQ, United Kingdom

**Keywords:** pangenomes, machine learning, evolution

## Abstract

Pangenomes exhibit remarkable variability in many prokaryotic species, much of which is maintained through the processes of horizontal gene transfer and gene loss. Repeated acquisitions of near-identical homologs can easily be observed across pangenomes, leading to the question of whether these parallel events potentiate similar evolutionary trajectories, or whether the remarkably different genetic backgrounds of the recipients mean that postacquisition evolutionary trajectories end up being quite different. In this study, we present a machine learning method that predicts the presence or absence of genes in the *Escherichia coli* pangenome based on complex patterns of the presence or absence of other accessory genes within a genome. Our analysis leverages the repeated transfer of genes through the *E. coli* pangenome to observe patterns of repeated evolution following similar events. We find that the presence or absence of a substantial set of genes is highly predictable from other genes alone, indicating that selection potentiates and maintains gene–gene co-occurrence and avoidance relationships deterministically over long-term bacterial evolution and is robust to differences in host evolutionary history. We propose that at least part of the pangenome can be understood as a set of genes with relationships that govern their likely cohabitants, analogous to an ecosystem’s set of interacting organisms. Our findings indicate that intragenomic gene fitness effects may be key drivers of prokaryotic evolution, influencing the repeated emergence of complex gene–gene relationships across the pangenome.

## Significance

Different strains of the same prokaryotic species often show significant variation in gene content. Whether this variation is due to genetic drift or selection is not well understood. If the latter, we expect sets of genes to be consistently and repeatedly gained or lost together, or sequentially. We used machine learning to predict the presence of variable genes in a large set of *Escherichia coli* strains, using other variable genes as predictors. We find a large proportion of genes are predictable, suggesting selection plays a role in their acquisition, loss, and maintenance. We show that some genes are consistently associated with the presence or absence of others. These results have implications for understanding evolutionary dynamics in prokaryotic genomes.

Evolution by horizontal gene transfer (HGT) and differential loss causes remarkable variation in gene content in bacterial genomes, both within and between populations (1–5). Genes present in all genomes in a collection constitute the core genome, while genes that are found only in some lineages are accessory genes. The union of these two sets makes up the pangenome. Intraspecific HGT, mediated by plasmids, phage, and transformation account for most gene transfers into a genome. Though there has been some disagreement on the relative influences of random drift and natural selection on structuring pangenomes, it is understood that the presence or absence of specific genes (genetic background) can influence the presence or absence of others (6–8). Consequently, the content of every contemporary prokaryotic genome is an outcome of its history of vertical and horizontal gene transmission and has emerged via a combination of internal (intragenomic) and external (ecological) fitness effects (9) in addition to stochastic, nonadaptive evolution (genetic drift).

It is also unclear how evolutionary responses, say, to the acquisition of a gene by HGT, are sensitive, or robust, to differences in evolutionary history. In his book, “Wonderful Life: The Burgess Shale and the History of Nature” (10), Stephen J. Gould set out a thought experiment where the “tape of evolution” could be replayed from any point in history. He suggested that since evolutionary paths depend on unpredictable events, if we could replay history, it would not result in the same outcome each time. Many recent studies have suggested this view is too rigid. Experiments designed to mimic replaying of the tape, such as parallel evolution experiments, have suggested that historical contingency does indeed have an effect but that some aspects of evolution are deterministic—i.e., they are likely to happen each time we replay the tape (11–15). Until now, it has not been obvious how, or even if, the contingency-deterministic question relates to prokaryote genome evolution. In prokaryote pangenome evolution, repeated HGT can introduce homologs of the same gene family into divergent genomes that contain unique but overlapping sets of genes. The incorporation of these genes into different genetic backgrounds allows us to address the contingency-determinism question through retrospective analysis of the subsequent outcomes. We identify a deterministic outcome if all, or most, recipient lineages evolve in similar ways after gene acquisition, while the alternative is that prior events—divergence in gene content of the recipient genomes—would play the more important role, and postacquisition evolution of the different lineages would therefore be markedly different.

In the context of this study, we define a deterministic evolutionary trajectory as the acquisition of a gene that in turn potentiates the acquisition, avoidance, retention, or loss of one or more other genes. In other words, certain evolutionary outcomes are highly likely, thanks to the influence of intragenomic selection on genotypes. Repeated acquisition and loss of a gene, while necessary, is insufficient to imply deterministic evolution. Hallmarks of determinism would include the emergence of repeated biases in gene content, including the selective recruitment of another gene, or selective loss of another gene, following horizontal transfer. Owing to the prominent role of stochastic processes in evolution, it is unlikely that gene content evolution is entirely deterministic or entirely driven by contingency, but rather it falls somewhere on the spectrum between both extremes. The question is which end of the spectrum is closest. Today, several thousand complete prokaryotic genomes are available, providing enough data to address the issue. Therefore, we can ask whether a gene’s presence or absence in a genome is predictable, based solely on the gene content of the rest of a genome. This would imply deterministic evolution. Alternatively, if gene presence or absence is not predictable, it is because its presence is either contingent on unaccounted differences in evolutionary history or is solely driven by genetic drift. We acknowledge that HGT and loss is not the only way that gene content can evolve in a pangenome. Other forms of mutation including single nucleotide substitutions are clearly influential in bacterial evolution (see ref. 16), and this may affect the ways in which genes co-occur or avoid each other. However, here we focus exclusively on gene gain, occurring usually through transfer, and loss.

Several software programs have been developed to find coevolving gene pairs and to infer coevolving modules (17–22). However, gene presence or absence in a genome may be influenced by a mix of positive and negative intragenomic effects beyond just pairwise correlations. To incorporate these more complex and subtle patterns, we used a Random Forest approach (23). Random Forests aggregate information from individual decision trees, which themselves summarise the conjunction of features not just pairwise comparisons, that lead to predictions of gene presence or absence.

A Random Forest approach can assess whether inferences are generalisable. The model that we use to predict gene presence or absence is parameterised on a training dataset and evaluated on a test dataset (24). If the model built using the training dataset does not describe the patterns found in the test set, it is probable that the pattern is an artefact of the training set, and the model should be considered inadequate. However, if the model makes accurate predictions in the test set, it appears to describe general properties of the entire dataset. Finally, Random Forest models make predictions in a directed manner, where one gene might predict the presence or absence of another, meaning that we can say whether a gene is predictable, and if so, we can also identify its predictors.

In this paper, we demonstrate that a substantial proportion of *Escherichia coli* accessory genes can be predicted by the other genes present. *E. coli* has a large accessory genome (25, 26) and occupies a wide range of niches (27). The *E. coli* pangenome has evolved divergent gene content over time—so much so, that a gene that is horizontally transferred from one *E. coli* to another will often find itself in a considerably different ensemble genetic background. We have analysed the predictability of gene content evolution following the repeated transfer of genes into these diverse genetic backgrounds. This is a natural equivalent of what Blount, Lenski, and Losos called a “historical difference experiment” (11). We have typified the effects of accessory genes’ presence on the presence or absence of other genes into three categories typically used by macroecologists to describe interactions between species. McInerney defined mutualism as a situation where two or more genes benefit from the association (9). Here, we define putative mutualism as two genes predicting the presence of one another and each gene similarly influencing the likelihood of the other’s occurrence. This could be due to a genuine beneficial relationship between the two genes. However, they might also both benefit from a common factor, which doesn’t necessarily have to be another gene. Commensalism refers to the situation where one gene strongly depends on the presence of another, but the reverse dependence is much weaker or nonexistent. Competition is when two genes appear to avoid being in the same genome. Note that we are not attributing specific behaviours to genes; these categories merely serve to describe observed patterns.

## Materials and Methods

*E. coli* genomes were downloaded from the National Center for Biotechnology Information (NCBI) genome database (28) using the NCBI command line utility “datasets” 12.17.2 accessed 01/05/2022. All annotated *E. coli* genomes were downloaded provided that they were of the highest completeness level (complete). If a genome had been assembled by both the GenBank and RefSeq methods, the GenBank (GCA) annotation was maintained; however, if only a RefSeq (GCF) annotation was in the database, this was retained. The final dataset consisted of 2,341 genomes. The full list of accession numbers is included in *SI Appendix*, *Supplementary List 1*.

Genomes were re-annotated with PROKKA version 1.14.6 (29), employing the “bacteria” annotation mode. We applied an E-value threshold of 10^−9^ and mandated a minimum query coverage of 80% for assigning functional annotations. The *E. coli* pangenome was inferred using Panaroo version 1.2.9 (30) with the mode set to sensitive. It was important to include rare genes, owing to the possibility that they would have important effects on the prediction of other genes.

The gene presence–absence matrix produced by Panaroo was processed so that genes present in more than 99%, or less than 1% of genomes were removed. The matrix was further modified by converting gene names to “1” and empty fields to “0” so that 1 represented presence and 0 represented absence. In addition, genomes with identical patterns of gene presence and absence were collapsed into the same vector. Genes with identical presence-absence patterns (PAPs) across our genome sample were also collapsed into gene family groups (*SI Appendix*, Table S1). Essentially, this meant that both the features and predicted variable in these analyses are PAPs rather than genes per se, most of which were represented by only one gene but some of which were represented by multiple genes. The list of genomes with nonunique gene repertoires is found in *SI Appendix*, Table S2.

To minimise the impact of phylogeny on understanding predictability, repeatability, and contingency, we impose the requirement that we only study genes whose distribution is not “clumped” on one or a few clades. A backbone phylogeny was inferred in order to evaluate the distribution of gene content across the tree. Alignments of universal single-copy genes were constructed using MAFFT version 7.490 (31). The resulting 337 gene alignments were concatenated to form a superalignment and a maximum likelihood phylogeny was reconstructed using IQTree 2.2.0 (32) with extended model selection (33) and 1,000 ultrafast bootstrap replicates (34). The tree was rooted at the midpoint. To assess the distribution of each gene on this tree, we calculated Fitz and Purvis’ D statistic (35), retaining only those predicted genes with a D statistic greater than zero, though source nodes could still have D < 0. To contextualise the meaning of the D statistic, we also calculated the parsimony score for each gene using PAUP v4.0a (36). This metric represents the minimum number of times a gene has shifted from present to absent or absent to present along the phylogeny. Filtration of the edges was carried out using Sqlite3 (37). In practice, we only study genes that have changed character state from present to absent or vice versa on the tree at least 8 times and where D is >0.

A model of gene presence or absence was inferred using Random Forests. Prediction models for each PAP were calculated separately, meaning that the number of times that our Random Forests were trained was equal to the number of PAPs in the processed matrix. For each PAP being predicted, the genomes were randomly split into a training set, equal to 75% of the genomes and a test set of the remaining 25%. These sets were stratified by the gene presence–absence state being predicted, meaning that the proportions of genomes with any given gene being present or absent remained approximately the same in the training and the test datasets. These test and training datasets were assigned independently during the prediction of every PAP in every analysis. Decision trees were then generated by taking a random sample of genes with size equal to the square root of the number of genes in the dataset and the gene that most evenly split the training set out of this subset formed the first node in the tree (according to ref. 38). This process was repeated on each side of each decision node until either the maximum depth was reached or the remaining sample of genomes all had the same state for the gene in question. The impact of varying the number of trees produced and their maximum depth was empirically assessed based on the average effectiveness of the analysis in predicting the presence or absence of each gene (*SI Appendix*, Fig. S1). We chose the maximum depth and number of trees above which the performance of the models on the test set did not increase substantially. For the analyses in this manuscript, including those where the dataset was downsampled, we used 1,000 trees with a maximum depth of 16 nodes per tree.

For each gene (or set of genes with the same PAP), a prediction of either its presence or its absence was obtained for each genome in the test set according to the model generated using the training set. For each gene, four performance metrics were taken. These were Accuracy, Precision, Recall, and F1 score, all defined according to ref. 39. As our classification algorithm has two classes, we recorded a version of recall, precision, and F1 score separately for both the gene presence and for the gene absence classes.

In addition to performance statistics, the Gini importance (40) of each gene in predicting each other gene was added to an *n* by *n* matrix where *n* is the number of PAPs in the dataset. Here, the Gini importance is the contribution of a predictor PAP in separating the dataset into genomes where the test gene is present and those where it is absent averaged over all trees in the Random Forest. This Gini importance value can be used as a measure of the strength of the impact of the predictor gene on the presence/absence state being predicted. This statistic is appropriate when the predictor variables have two classes, as is the case here (41). All machine learning algorithms were implemented using the scikit-learn Python module version 1.0.1 (38). Code is available at https://github.com/alanbeavan/pangenome_rf (*SI Appendix*, Fig. S2).

In principle, it is possible that a gene’s presence may be predictable purely due to coincidental correlations arising by random transfer and loss across the phylogeny. The number of predicted genes occurring in this way can be thought of as a baseline level of false positives. We simulated the presence or absence of genes on each tip of the tree according to an nonreversible All rates Different model which allows for a different rate of 1-to-0 and 0-to-1 transitions (see ref. 42). For each gene family, the substitution matrix was derived from the gene presence and absence patterns observed across the phylogeny. This was achieved using the “fitDiscrete” function from the “geiger” R package (43). Following this, a presence and absence pattern for each gene was simulated using the “simulate_mk_model” function, which is part of the castor R library (44). For simulation, the probability of the gene being present at the root of the tree was equal to the root probabilities inferred by IQTree (32). We employed ancestral state reconstruction and extended model selection and used the phylogeny inferred in this study as a fixed topology. Simulations were conducted 100 times and then analyzed in a manner similar to the empirical data. However, for computational efficiency, only those Presence-Absence Patterns (PAPs) with a high F1 score (as determined in the post-processing of the network) had their D statistic estimated.

### Postprocessing the Network.

Initially, all relationships between PAPs with a GINI importance of less than 0.01 were removed from each network examined. This threshold is arbitrary, chosen to restrict the number of gene–gene relationships to the most highly ranked, a number that could be visualised.

Co-occurrence was distinguished from avoidance according to the effect of the predictor gene on the likelihood of the predicted gene’s presence. If the frequency of the target was higher when the source was present, the relationship was set as “co-occurrence.” If it was lower, then the relationship was flagged as “avoidance.” To ensure that our model only contained strongly predicted relationships, we kept only those edges where the target node was classified as accurate, which here means that the F1 score for both classes (present and absent) was greater than or equal to 90% in the test set.

Artefactual splitting of de facto gene families can, in principle, take place during pangenome construction. Therefore, using BLASTn (45, 46), we subjected the sequences of each gene family involved in any apparent avoidance relationship to a comparison with the gene family they avoid. A total of 563 gene families (before filtering for performance and D) featured at least one sequence that was identical to a sequence placed in a different family. Gene families with this property were therefore removed from our results. After this step, any two sequences from avoidant gene families never share identity of at least 50% of their nucleotides or produce a significant BLAST hit with an associated E-value < 10.

### Functional Classification of Genes and Enrichment Analysis.

Eggnog mapper version 2.1.8 (47) was used to assign gene ontology terms [go-basic release 2022-07-01 (48, 49)], and Kyoto Encyclopedia of Genes and Genomes (KEGG) pathway (50, 51) terms for each gene included in this analysis. This applied to genes with a genome occupancy ranging between 1% and 99%. Diamond-BLAST (52) was used to search the eggnog mapper database for sequences with an E-value <10^−5^. For functional enrichment analysis, the program used was find_enrichment.py which is part of the GOATOOLS Python library (53).

### Calculating Physical Linkage between Genes.

The physical distance between two genes was measured in the number of genes separating them, rather than base pairs. The position of each gene of the pair was extracted in each genome from the Panaroo output (30). For each pair of genes, the distance separating them was defined as the minimum distance either going clockwise or going anticlockwise around the circular chromosome. Genes present on different elements (e.g., chromosome and plasmid) were also enumerated in this way.

### Categorising Accessory Gene Dynamics.

We placed observations of gene associations into three mutually exclusive categories. The first category was putative mutualisms, where two or more genes predict each other’s presence and the strength of the prediction was similar in both directions, with the F1 statistic greater than or equal to 0.9 and the D statistic is greater than zero. For commensalisms where the presence of one gene was highly dependent on the presence of another, but the reciprocal dependency was either far weaker or nonexistent, the more abundant, or “host gene,” must have been present in almost all (>= 99%) genomes where the putative “commensal” was present. Additionally, the proportion of genomes without the commensal where the host was present must have been at least 20% of the proportion of genomes in the full dataset containing the host. That is, if the host gene occupied 50% of the genomes in the full dataset, it must have been present in 10% of the genomes where the commensal gene was absent. Otherwise, we did not classify a relationship as putatively commensal. If a gene–gene association was classified as commensal, it could not be classified as putatively mutualistic. Finally, we considered two genes to be putatively in competition or antagonistic of one another when the absence of one gene strongly predicted the presence of the other or vice versa, i.e., any pair of genes where the predicted gene passed the thresholds described above and was found at a lower frequency when the predictor gene was present.

### Data Visualisation.

**Fig. 1.**
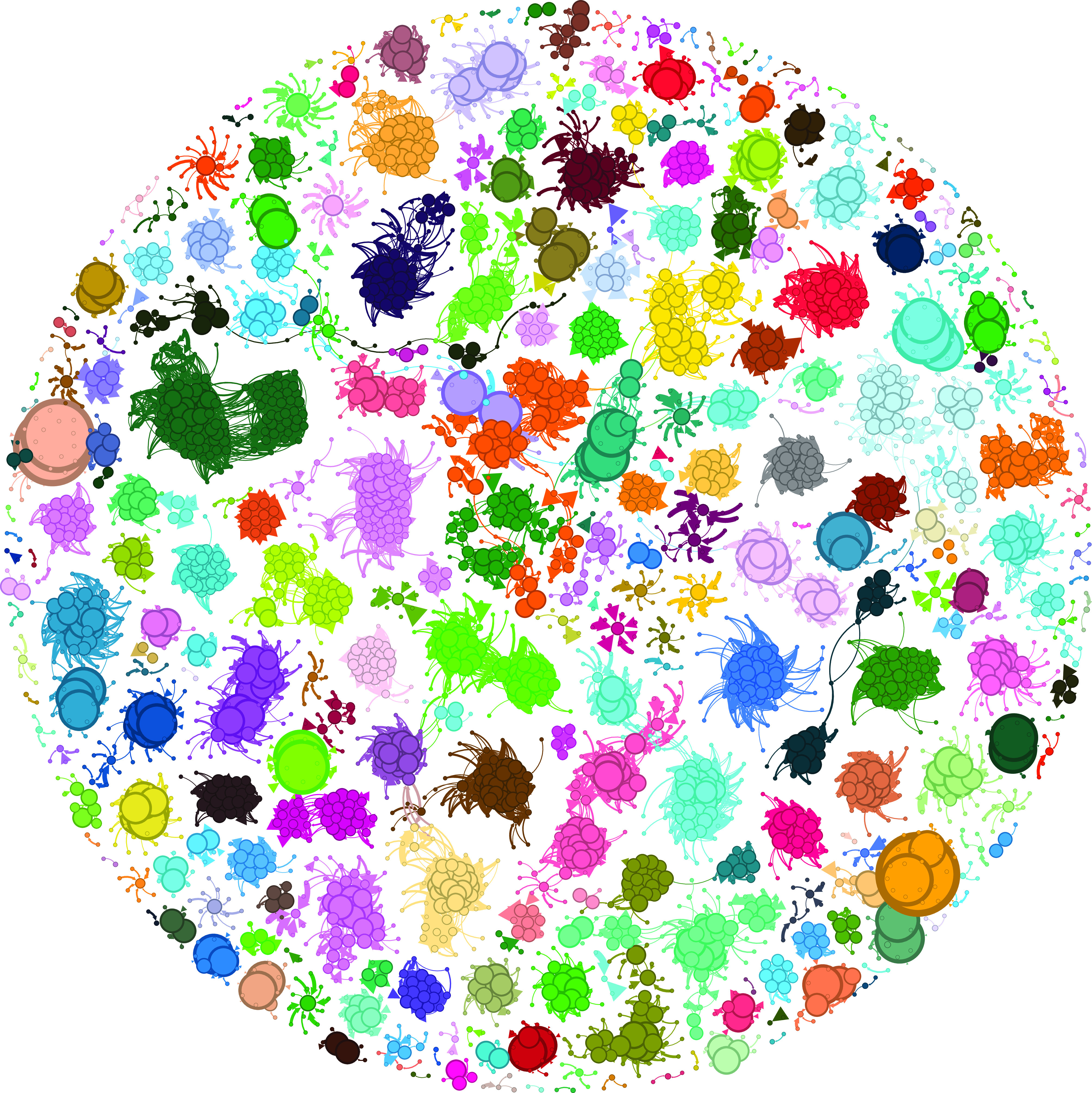
The coincident relationships of predictable genes and their predictors. The nodes are gene families, or groups of gene families with the same PAP, and the edges are coincidence relationships with the arrow pointing at the node whose presence is predicted by the other. Edge thickness is proportional to the GINI importance value scaling linearly from the thinnest at 0.01 to the thickest at 0.062, while node size is proportional to the PageRank (56) value for that node. The PageRank algorithm has been applied here to evaluate the relative importance of each node within the network. A larger node size indicates a higher PageRank score, suggesting that the node has more influence or is more central in the network. Node size scales linearly from the smallest with a PageRank of 0.000043 to the largest with 0.002945. This visualisation aids in quickly identifying key nodes that play pivotal roles in the connectivity and flow of the network. Node colour indicates community as identified by the Louvain algorithm (57). This figure can be thought of as a high-level summary of the results of this analysis, and attention should be paid to the number of nodes in a community, the discrepancy in the size of nodes, and the number of edges emerging from nodes, in this figure, source nodes can have a D score < 0. For a version of this figure with these nodes removed, see *SI Appendix*, Fig. S8.

Gephi v0.10 (54) was used to visualise networks. Network layout was achieved using the Fruchterman–Reingold layout algorithm (55). Node ranking was assigned according to the PageRank (56) algorithm weighted by Gini importance of each incoming arc. A considerable amount of variation was observed in the weight ascribed to the nodes on this network when we employed the PageRank approach to ascertain node importance. Specifically, to attach a ranking to a node, the algorithm combines the number of incoming arcs, the weight of the arcs, and the importance of the source nodes (56). Node size in Fig. 1 is proportional to node rank, as judged by the PageRank algorithm. Other graphics were produced using custom Python or R scripts.

## Results

### A Substantial Subset of Accessory Genes in *E. coli* Can Be Predicted Accurately.

The *E. coli* pangenome inferred from 2,241 genomes in this study contained accessory gene families with 12,840 unique PAPs that were present in more than 1% and less than 99% of genomes and were hence included in this study. 56,579 gene families were inferred by Panaroo but 28,774 genes were not included in the analysis because they were present or absent in over 99% of genomes. These were mostly very rare genes. Of the remaining 27,806 genes, 19,137 had a presence–absence pattern that was shared by at least one other gene and were hence collapsed into 4,172 presence–absence patterns in addition to 8,668 genes with unique distributions. The presence or absence of 3,922 (30.5%) PAPs could be accurately predicted (both F scores >= 0.9) in the test set after the Random Forest model had been trained. From this accurately predicted dataset, a total of 2,144 (54.7%) had an associated D-statistic greater than 0, meaning that they were distributed widely on the tree. The remaining 1,778 PAPs were “clumped” on the tree, and therefore, it is more difficult to ascribe causality to their association, when a very good explanation might be that they were simply acquired at more or less the same time and have been vertically inherited together since then. *SI Appendix*, Fig. S3 shows that although the D score is not directly proportional to parsimony score, it correlates strongly, meaning that all 2,144 PAPs with a D score of greater than or equal to zero also had a parsimony score of at least eight though most had a much higher score (*SI Appendix*, Fig. S3). This means that we have only examined predicted genes which have been acquired and/or lost at least 8 times across the pangenome, and furthermore, we insist that their distribution is widespread and not localised (35). We focus on this set of 2,144 PAPs because they manifest a broad, patchy distribution across the phylogeny, stemming from a combination of lateral gene transfer and loss, and we can accurately predict their presence or absence based on the other genes present in the genome.

To evaluate whether the presence–absence matrix of 12,840 uniquely distributed PAPs is no better structured than random expectation based on the underlying phylogeny and gene gain and loss rates in this study, we compared results from the original data to those simulated based on inferred transition rate matrices. Simulated datasets were analysed in the same way as the empirical data. Treating this as our null hypothesis, we can evaluate the extent to which, even after filtering by the D statistic, the predictability of a gene’s presence or absence can be explained by chance. In each simulated dataset, several genes pass the F1 score thresholds but the majority of these can be explained by a low D score and are hence removed from the set of accurately predicted genes. The number of genes that successfully pass both thresholds is between 1.0% and 1.7%, which can be thought of as a false discovery rate. The empirical analysis yielded 16.7% of genes accurately predicted with D > 0 (*SI Appendix*, Fig. S4). Accordingly, we can reject the hypothesis that these empirical observations of associations have arisen solely due to chance in our dataset or that the structure of the pangenome dataset has no more gene–gene correlations than the structure of randomly assembled data.

In principle, we would expect the number of accurate predictions to increase with increasing quantity of data, provided that the predictions being made are not artefactual. Hence, if downsampling the dataset results in a decrease in predictions being made accurately, it would be reasonable to infer that the addition of more data would result in more accurate predictions being made. Therefore, we carried out a sensitivity analysis on dataset size. We randomly eliminated 50%, 75%, 90%, and 95% of the genomes in the dataset and then repeated our Random Forest prediction 10 times per dataset. In each case, reducing the number of genomes substantially and significantly reduced the number of PAPs that were accurately predicted, while having a much smaller effect on the number of total PAPs that could be analysed. For example, the average number of accurately predicted PAPs, over 10 repeated analyses after filtering PAPs with D score < 0, using 50% of the genomes was 1,650/12,642 (13.1%) compared with the 2,144/12,840 (16.7%) in our full analysis. When only 5% of genomes were included in the study, an average of 713/11,644 (6.1%) PAPs were predicted accurately (*SI Appendix*, Fig. S5). This suggests that predictions would be likely to improve with the addition of more genomes.

The links between the 2,144 predictable PAPs, were used to construct a network with 33,426 edges featuring all well-predicted target nodes and their predictors (Fig. 1). This network consisted of 243 connected components ranging in size from 2 to 248 nodes, featuring both coincident and avoidance edges sensu ref. 19. By considering only the coincident relationships (33,138 out of 33,426 edges), we found 240 connected components containing between 2 and 244 nodes. Taking only avoidance relationships, 28 connected components were generated with a range from 2 to 22 nodes in size. As nonunique gene patterns are collapsed into one entity, both in the analysis and presentation of results, some of the nodes represent multiple genes. Out of the well-predicted PAPs, 827 patterns were observed in more than one gene. In total, independent of whether they are well predicted by our Random Forest model or not, 19,137 genes had nonunique PAPs and were collapsed into 4,172 patterns that were then used both as features for prediction and as patterns to predict.

Owing to the stochastic nature of the Random Forest approach, we repeated the analysis 100 times, each time splitting the data into training and test sets differently. Out of the 12,840 accessory genes with unique PAPs analysed, 5,020 were never classified as predictable, 939 were always classified as predictable, 4,395 were classified as predictable in only some analyses (*SI Appendix*, Fig. S6), and the remaining 2,486 had a D score < 0. To understand what makes a gene’s presence or absence predictable, we compared the 939 PAPs that were always well predicted the least predictable gene families in the dataset, which we defined as the set of genes that never passed our thresholds of predictability in any of the 100 iterations of the RF algorithm. To ensure a balanced comparison, we took the 939 PAPs with the lowest average combined F1 score over the 100 repeats to generate two equally sized datasets representing the most and least predictable genes.

Following the functional annotation of genes using EGGNOG mapper (47), analyses of enrichment of Gene Ontology (48), and KEGG (50) pathways were performed. Using *P*-values that were corrected by false discovery rate, the set of most predictable genes were not enriched in any function, cellular location, or process and were not enriched in any gene ontology terms in either biological process, molecular function, or cellular compartment. Additionally, only two KEGG pathway terms were enriched in the consistently predictable genes. These were “bacterial secretion system” and “flagellar assembly.”

Among the 939 least predictable genes, 1 Biological Process term was enriched, and 22 were underrepresented, while for the cellular compartment terms, 0 were enriched, and 14 were underrepresented, and for molecular function, there were no enriched terms, while 4 were underrepresented. No KEGG pathway was found to be enriched or purified in this dataset after FDR correction. In total, 1 GO term was enriched in the low predictability set of genes, while 40 GO terms were significantly underrepresented (*SI Appendix*, Table S3).

Given that the most predictable genes appear to contribute to a range of functions, we investigated the extent to which physical linkage determined their predictability. We compared the positions of the genes that shared an association and measured the distance between them (in numbers of genes rather than base pairs) as inferred by Panaroo. From these distances, linkage clearly plays an important role in the association of two genes (*SI Appendix*, Fig. S7). In the set of genes with the most accurate predictions, 68.7% of pairs of associated genes within the same genome are found to be separated by a distance of 10 genes or fewer. However, even in the most predictable set of genes, there are several occurrences of genes that are not closely physically linked with the genes that they are coincident with (10.4% of pairs of genes were separated by at least 21 genes), so it cannot be the only factor at play. In addition to pairs of coincident genes on the same genomic element, there were 17,714 coincident pairs of genes where one is featured on a chromosome and another on a plasmid in the same genome.

### The pangenome as an Ecosystem.

Almost exclusively, the debate surrounding accessory genome evolution has been framed in terms of the “usefulness” of genes to their host or the quality of the fit between a particular gene function and the external environment in which the host is found. However, genes also have effects on one another, requiring us to consider higher level conceptualisation of the dynamics of gene gains, losses, and the intrinsic and extrinsic forces that shape pangenome evolution. Tansley (58) developed the theory of the ecosystem, progressing the study of ecology from a focus on individuals to sets of interacting organisms. We here attempt to lay the groundwork to do the same in the context of pangenomes by characterising gene–gene relationships according to their patterns of occurrence. This aids our understanding of gene content evolution by not only showing the extent of intragenomic influences on gene fitness but also how complex relationships can influence gene content evolution both on the scale of the whole accessory genome and for specific examples of sets of genes (9).

**Fig. 2.**
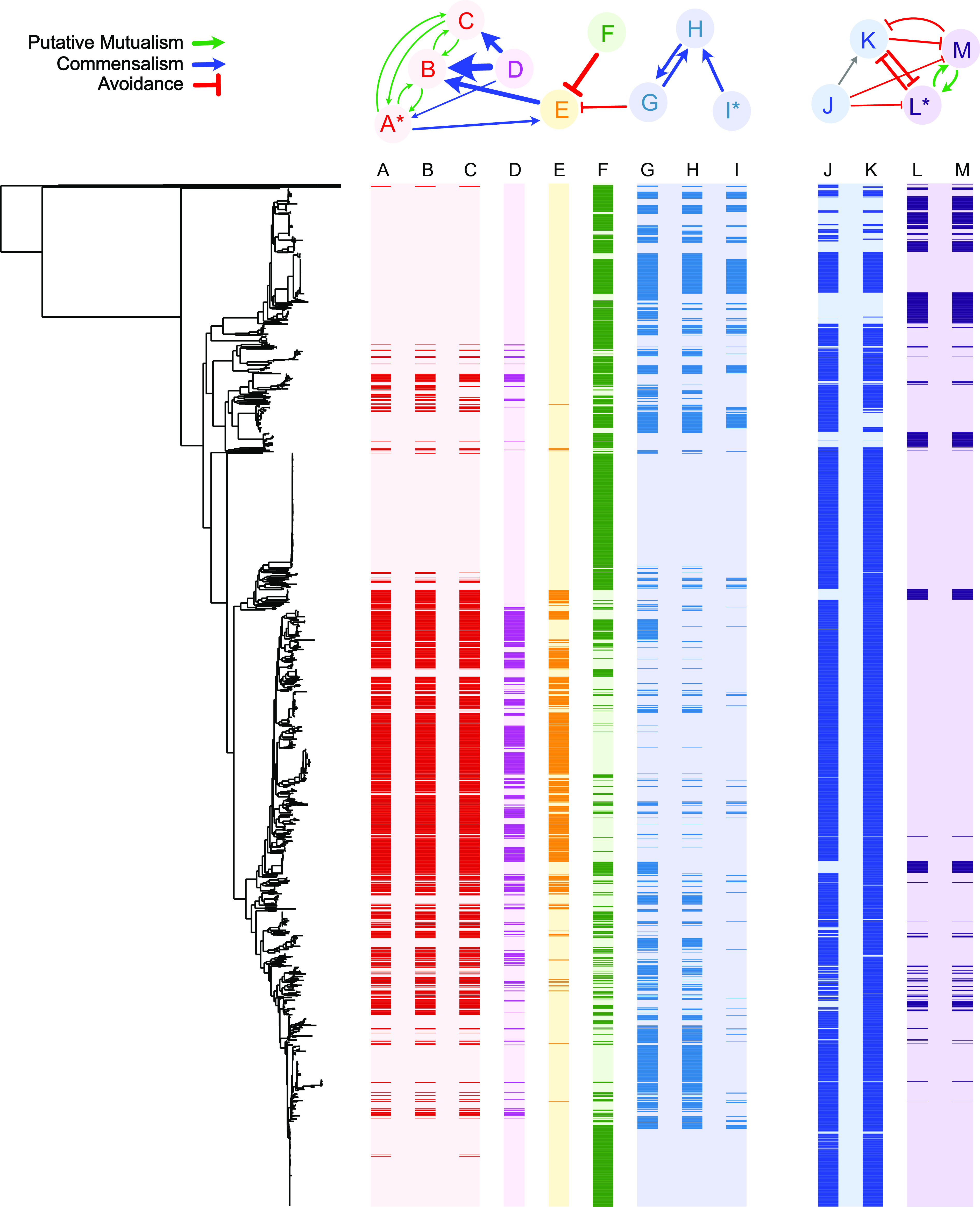
Relationships between selected presence–absence patterns in the *E. coli* pangenome. On the top are a network of nodes that represent the presence–absence patterns of the columns directly beneath, as well as the connections between the nodes that represent significant co-occurrence and avoidance relationships. Below left, the backbone phylogeny of the genomes in this study is positioned such that the rows of the heatmap to its right represent the presence or absence of nodes according to the label above each column. In each, the colour of presence is indicated by the colour of the text labels in the network and the colour of absence by the background colour of the node. Columns are coloured differently for differentiation rather than their properties. The colours of the arrows in the network indicate the type of association inferred. The figure we produced was created with the help of a modified version of the program ‘roary_plots.py’, which is a component of the Roary suite of tools. (59). The gene annotations are as follows. Node A: a set of genes with identical presence–absence patterns including *farR*, *hpcG*, *ttuB*, *hpcB*, *hpcE*, *hpcD*, *hpcH_2*, *iolA*, and *hpaB*. Node B: *hpaC*. Node C: *rhaR_2*. Node D: group_39613 (not functionally annotated). Node E: *pac*. Node F: *symE*. Node G: group_13180 (not functionally annotated). Node H: group_19718 (not functionally annotated). Node I: A set of genes with identical presence–absence patterns including group_19717, *hsdM*, and *mrr* (see *SI Appendix*, Table S4 for full annotations). Node J: *lgoT*. Node K: mdtM. Node L: A set of genes with identical presence–absence patterns including group_24769 (*SiaP*), *siaT*, and *nhaK*. Node M: *dctM*:*siaM* (see *SI Appendix*, Table S5 for full annotations).

We investigated three signature relationships within the *E. coli* pangenome that we term putative mutualisms, commensalisms, and competition. We have illustrated these relationships using a small subset of the data, outlined in Fig. 2. By far, the most frequent category of relationship is a putative mutualistic coincident relationship where the joint presence of a pair of genes in genomes is significantly higher than expected from their overall frequency in the dataset. We recovered 20,915 putatively mutualistic coincident relationships out of our total of 33,138 inferred relationships (Fig. 2 A–C). In our network analysis, we observed a total of 2,073 instances of commensal relationships. In these relationships, one gene, typically the less abundant of the pair, generally depends on the other, while the reverse dependency is much weaker or nonexistent. This was determined as outlined in the Materials and Methods section. Commensal relationships can be seen in Fig. 2 between PAP pairs DA, DB, DC, EA, EB, and IH. Finally, competition relationships, which are defined as pairs of genes that avoid each other, were observed in 288 cases.

Twenty connected components in our graph consist of genes that show both competition and coincident relationships in which two coincident gene sets have a reduced probability of being in the same genome at the same time (Fig. 2). Although the set of competition relationships is the smallest of our three categories, it represents the interesting situation where one gene makes a genome much less hospitable to another. In Fig. 2, we see that nodes F and G both predict the absence of node E. The reciprocal is not seen, though an analysis of the importances shows that the reciprocal relationship for E and F is just below our cutoff value (0.00925, when the cutoff was 0.01). To ensure avoidance relationships that we identified are genuine, we carried out a post hoc analysis of the avoiding gene pairs, and none of the genes share a sequence identity of at least 50% between each family or an E value < 10.

In Fig. 2, we outline a set of PAPs that represent one or more gene families, that predict the presence or absence of at least one other gene family. In the cases outlined, none are plasmid borne. In addition to being good examples of putative mutualism, commensalism, and competition, the genes that manifest these PAPs are also of translational importance. For example, PAP E is the “*pac*” gene. During a cell’s response to penicillin, the Pac protein catalyses the hydrolysis of penicillin, forming six-aminopenicillanate, which is also important in the manufacture of synthetic penicillins (60, 61). The presence or absence of the encoding gene is predicted accurately in *E. coli* genomes using our Random Forest approach. Using parsimony reconstruction, we estimate that there have been at least 72 changes from present to absent or absent to present for this gene family across the phylogeny. Furthermore, the analysis of its distribution across the tree shows that it has a D score of > 0. Three other PAPs in our dataset are strongly predictive of the presence or absence of *pac*. These are PAPs G [group_13180 (not functionally annotated)] and F (*symE*), which are single gene families, and their presence strongly predicts the absence of *pac*, and the set of genes indicated by node A (*farR*, *hpcG*, *ttuB*, *hpcB*, *hpcE*, *hpcD*, *hpcH_2*, *iolA*, and *hpaB*) that, conversely, strongly predict the presence of *pac*. Of these three PAPs, perhaps the most striking relationship is the avoidant relationship between PAP F, the gene *symE*, which is more abundantly represented throughout the dataset, yet it displays a genome occupancy pattern that is completely mutually exclusive with the *pac* gene. There are no genomes where both genes coexist, although there are several where neither of the genes are present. *symE* has a parsimony score of 167 and is a translational repressor associated with the SOS response (62).

We also outline the predictive relationships between four other PAPs (Fig. 2 J–M). To annotate these genes, a representative protein sequence for each gene family was compared with the NCBI nonredundant protein database using BLASTP. PAP J is annotated as *lgoT*, which is an MFS Transporter. PAP K is annotated as *mdtM*, a multidrug efflux MFS transporter. PAP L is a collection of three distinct families that co-occur perfectly. PAP L includes *nhaK*, a Na+/H+ antiporter, *siaP*, which is a C4-dicarboxylate TRAP transporter substrate-binding protein, and *siaT* which is a TRAP transporter small permease. PAP M is annotated as *dctM*:*siaM*, which is a TRAP transporter large permease. The presence of PAP J (*lgoT*) is predicted by the presence of PAP K (*mdtM*) (though the reverse is not true). Both PAP J (*lgoT*) and PAP K (*mdtM*) predict the absence of PAPs L and M (collectively, *nhaK*, *siaP*, *siaT,* and *dctM:siaM*), with all the genes in PAPs L and M predicting each other’s presence.

The most likely observation for any randomly picked genome from this study is that its genome will include either *lgoT* and *mdtM* (both MFS transporters) together (1,882 genomes) or *nhaK*, *siaP*, *siaT,* and *dctM*:*siaM* (a sodium ion/proton antiporter and parts of the TRAP transporter complex) together (213 genomes). While this canonical pattern of either one group or the other group is by far the most likely motif, the data are somewhat noisy, with mdtM co-occurring with at least one gene it normally avoids in 115 genomes. Similarly, the co-occurrence relationships are noisy, for example, there are 65 genomes that contain *lgoT* but not *mdtM* compared with the 1,882 that contain both. The most consistent pattern of avoidance is the relationship of *lgoT* with the three genes *siaP*, *siaT,* and *nhaK*, which never co-occur, though there are 23 genomes where none of these genes are present. This means that we have found evidence that a multidrug efflux transporter and a sodium–hydrogen ion antiporter strongly predict the absence of the other. Only eight genomes contain none of the genes depicted in Fig. 2 nodes J-L. Each gene in this set exhibits dynamic loss and gain, as indicated by their parsimony scores: lgoT (63), mdtM (43), siaP, siaT, and nhaK (57), and dctM:siaM (55). This pattern suggests that the relationships between these genes have evolved independently multiple times throughout evolutionary history.

## Discussion

A mechanistic explanation for prokaryotic pangenome origin and evolution is emerging (64, 65). Here, we specifically focussed on detecting pangenome-wide repeated and predictable patterns of evolution using a Random Forest approach. In effect, we have asked whether gene content evolution is predictable and whether within-species evolution is constrained by intragenomic forces. The benefit of the Random Forest approach is twofold. First, it allows us to test our models of gene presence and absence on an independent test set that is not used to generate the model. This means that genes can be classified not only according to the genes that influence their likelihood of occurrence but by whether their prediction is generalisable. This is a study that shows that gene presence or absence is predictable based only on other genes in the genome. Second, Random Forests can consider complex relationships as well as simple pairwise correlations. For example, a hypothetical gene A may predict the presence of gene B only in the absence of gene C. These two aspects of the method differentiate it from previous methods (17–22).

Though it was necessary to filter the dataset to remove rare and almost universal gene families (almost 50% of the accessory genome), we identified strong predictors for approximately 30% of the remaining gene families, roughly half of which could be explained by correlation with the phylogeny, leaving 16.7% of pangenome as both predictable and distributed widely across the species. While this leaves much of the accessory genome in a category of nonpredictable, given the current data and method of analysis, it must be viewed in the context that we have only used a single species pangenome and therefore might ask how the inclusion of additional species and datasets might lend additional predictive power. Given that downsampling our dataset reduced the proportion of gene families that could be well predicted, it is likely that the addition of more genomes would aid prediction, and the effect of broadening the taxon sampling would be of interest in future studies. The pattern of repetition and predictability that we observe across more than 2,044 gene families is compatible with a model of deterministic evolution and more difficult to reconcile with an evolutionary process dominated by contingent events. This compares with those gene families that we were not able to predict accurately for which we cannot rule out contingency on outside factors driving their evolution. Furthermore, given the way in which we have analysed the data, the reasons for observing such widespread predictability stem from intragenomic natural selection causing biases in the cohort of other genes that are acquired, retained, or avoided by a genome. Another possibility is that two (or more) genes are found together because they are both selectively beneficial in the same environment. Hence, when a lineage colonises this environment, both genes are selectively recruited independently. The reasons for co-occurrence and avoidance are largely speculative and would require complex experiments to decipher. Biased presence–absence patterns have been noted previously (19, 21, 22, 66).

Throughout this study, we have been using the heuristic that homology is closely related to functional similarity and that the effects that genes have on one another are due in some way to the encoded functions of the proteins. Furthermore, we assume that these functions remain constant over time, despite evolutionary changes in the gene sequences. This is of course unlikely to be completely accurate (67, 68). Our dataset almost certainly contains genes that are placed into the same family but have different functions and confer different fitness effects. This limitation would certainly weaken our ability to make accurate predictions, though only a small portion of the dataset has verified function. Indeed, mutation can change the function of genes without changing the gene family to which they are assigned (69, 70). It is outside the scope of this paper to assess the role of point mutation on the predictability of bacterial genome evolution, but we hope that this approach may be applied to aspects of genome evolution outside of HGT.

We note that a lot of gene families are not well predicted given the current dataset and method of analysis. Our dataset might be too small to supply enough power for statistical inference, or indeed, intragenomic fitness effects do not overcome random genetic drift. Downsampling the dataset produced a significant reduction in prediction power. While the outcome with significantly larger datasets is uncertain, the link between dataset size and predictive power suggests that increasing the dataset might enhance prediction accuracy. Future inclusion of other factors such as external environment, gene expression levels, protein interaction, phenotypic, or modification data may also aid prediction. Recently, the development of gene-specific evolutionary rate models has shown a significant level of metabolic predictability (71). Of course, a gene whose presence or absence is nonpredictable in the current analysis might continue to be nonpredictable in enormous datasets, precisely because it is not impacted by co-occurring genes.

We have taken great care to minimise the confounding effect of genome relatedness. By applying the D-score filter (35), all gene families in our predictive model have a history of being gained or lost at least eight times across the pangenome, and furthermore, the distribution of any gene family cannot be “clumped” or restricted to just one part of the backbone tree. This is not a perfect way to eliminate the effect of phylogeny on the associations, but by coupling this approach with a very high threshold for predictability, we end up with an approach that shows correlations that are not just because a pair of genes happen to be in the same clade. Furthermore, by simulating gene presence–absence on the tree we inferred, we observe that chance relationships can only account for ~1.5% of genes’ predictability. Stricter D score cutoffs would reduce this false discovery rate but at the expense of failing to identify some genes that are truly predictable. Similarly, decreasing the cutoff would identify a larger number of truly predictable genes but is likely to raise the false discovery rate.

We arbitrarily chose a conservative Gini importance cutoff of 0.01 though there is no standard procedure for choosing a cutoff. Gini importance is a measure of the reduction in ambiguity of the test variable (gene presence or absence), with each node that comprises the prediction variable (a predictor gene), averaged over the trees in the forest. A Gini importance of 0.01 means that the ambiguity of the predicted gene is reduced by an average of 1% in each tree (bearing in mind it will only be sampled in a subset of trees). Gini filtering limited the number of associations to the strongest 33,426 connecting 4,067 nodes, after filtering before filtering by model performance and D-statistic. A weaker cutoff value of 0.005, for example, would have included 70,581 edges connecting 6,830 nodes. The distribution of edge strengths suggests that the number of edges will increase exponentially as that threshold is reduced linearly (*SI Appendix*, Fig. S3). Similarly, we chose an arbitrary, conservative, cutoff of 0.9 for accuracy and F1 score for both classes. Applying an accuracy threshold of 80% and an F1 score threshold of 0.8 would have resulted in the inclusion of more than 70% additional genes and relationships. Under these criteria, the number of predictable genes with a D statistic greater than zero would have nearly doubled, increasing from 2,044 to 3,704.

Linkage clearly plays an important role in coordinating the cooccurrence of sets of genes, but it cannot explain all the results. Also, it is not clear whether linkage is the cause or a consequence of co-occurrence. According to the selfish operon theory (63, 72), we would expect two genes that provide a fitness benefit when found together in a genome to evolve to be physically closer together on the chromosome, so they are less likely to be separated via recombination events. The most likely explanation is that close linkage is both a cause and effect of being found together. However, in addition to gene family co-occurrence where linkage plays a role, there are thousands of examples of gene families cooccurring in a repeated manner where linkage clearly has not played a role throughout the evolution of the pangenome, either because the genes are separated by a long stretch of DNA or are on different genetic elements.

Genes avoiding being in the same genome is an established phenomenon, with Bruns et al. (73), for example, showing that within the genus *Salinospora* biosynthetic gene clusters encoding iron siderophores avoid one another. In that case, the clusters encoded iron chelators with very different structures, but near-identical chemical properties, and avoidance most likely stems from functional redundancy. In Fig. 2, we outline two situations where avoidance is apparent. In PAPs J-M, both sets of genes encode proton antiporters. It is not clear whether the two sets of genes can functionally complement one another, in which case avoidance might be caused by redundancy in the same way as the iron chelators in *Salinosopra*. It is also possible that the functioning of the two antiporters either results in toxic effects when present together or they are competing for the same cellular resources, which would obviously result in a reduction in fitness for the organism that possessed both sets of genes, compared with a close relative that contained only one set of genes. It is also possible that one of the sets of these genes is preferable to the other in some environments, but in others, the reverse is true. For example, multidrug resistance can be conferred by the presence of several sets of genes, none of which are functionally related but each of which is selected by the presence of different antimicrobial drugs used in a treatment. Indeed, ecology has been shown to be a major driver of HGT (74). The situation in Fig. 2 PAPs A-I is more enigmatic, with no obvious functional similarity between *pac* and *symE*. However, they are found separately in genomes far more often than expected, and furthermore, the appearance of one of these genes in a genome occurs simultaneously with the removal of the other and vice versa at least 30 times across the phylogeny (Fig. 2 PAPs E-F).

Using nomenclature and analogies from ecology, the diversity of motifs embedded in the Random Forest network analysis allows us to identify putative mutualistic, commensal, and competitive classes of relationship, (see ref. 9). In this sense, the pangenome exists as a broad ecosystem, with individual genomes acting as evolving localities, where genes can either potentiate or alternatively reduce the likelihood of the presence of another gene. Just like a macroecological system, we see groups of genes with strong reciprocal co-occurrence patterns which we call putative mutualisms. Sometimes, these are just pairs of genes, but they are often groups of more genes which all co-occur reciprocally. Focussing on putatively commensal relationships, we identify specific examples where one or more genes appear to make a genome more hospitable to another or several other genes. In these cases, the more abundant gene is highly likely to arrive in the genome first or simultaneously with the less abundant gene. We also see many cases where the arrival of a gene into a lineage is concomitant with the loss of another gene, and this pattern is repeated across the phylogeny. There are several possible underlying reasons causing genes to predict the presence or absence of other genes, including functioning in a common pathway or process, redundancy, physical linkage, and shared evolutionary advantage in a new environment.

Ecosystems are known to be dynamic; they also tend to be resilient and to be somewhat resistant to overall change (75). In our analysis of the *E.* coli pangenome, we see features that are consistent with this perspective. We see a very dynamic system of gene gains and losses; we see repetitive gains of the same cohorts of genes, and we see the establishment of sets of relationships that are persistent through time and across the phylogeny. Due to the diversity of the *E. coli* pangenome, each time a gene is recruited to a new genome, it finds itself in a different, and sometimes substantially different, genetic background. Nonetheless, we observe repeated, predictable patterns of evolution following a gene’s transfer.

With respect to Gould’s “replaying evolution’s tape” thought experiment, our results lead us to suggest that it is likely that rewinding the tape back to the start of *E. coli* evolution would still result in hundreds or thousands of predictable events taking place that are not contingent on those highly unlikely events unique to each replaying of the tape. It is doubtful that the exact same evolutionary trajectories would play out, but several motifs would emerge over time.

Other machine learning algorithms such as neural networks may also be able to improve predictions by finding more abstract or subtle patterns in the data. Employing these types of methodologies, combined with the identification of further predictor variables and their application to diverse, large datasets of bacteria, archaea, or eukaryotes, represents promising future directions for enhancing our understanding of pangenome evolution.

## Supplementary Material

Appendix 01 (PDF)

Dataset S01 (TXT)

Dataset S02 (CSV)

Dataset S03 (CSV)

## Data Availability

All study data are included in the article and/or supporting information.
