# Supplementary material for: Contingency, repeatability, and predictability in the evolution of a prokaryotic pangenome
Source: Proc Natl Acad Sci U S A. 2023 Dec 26;121(1):e2304934120. doi: 10.1073/pnas.2304934120 (PMC10769857; doi:10.1073/pnas.2304934120)
Supplement: Supplementary file 1 — Appendix 01 (PDF) [file pnas.2304934120.sapp.pdf]

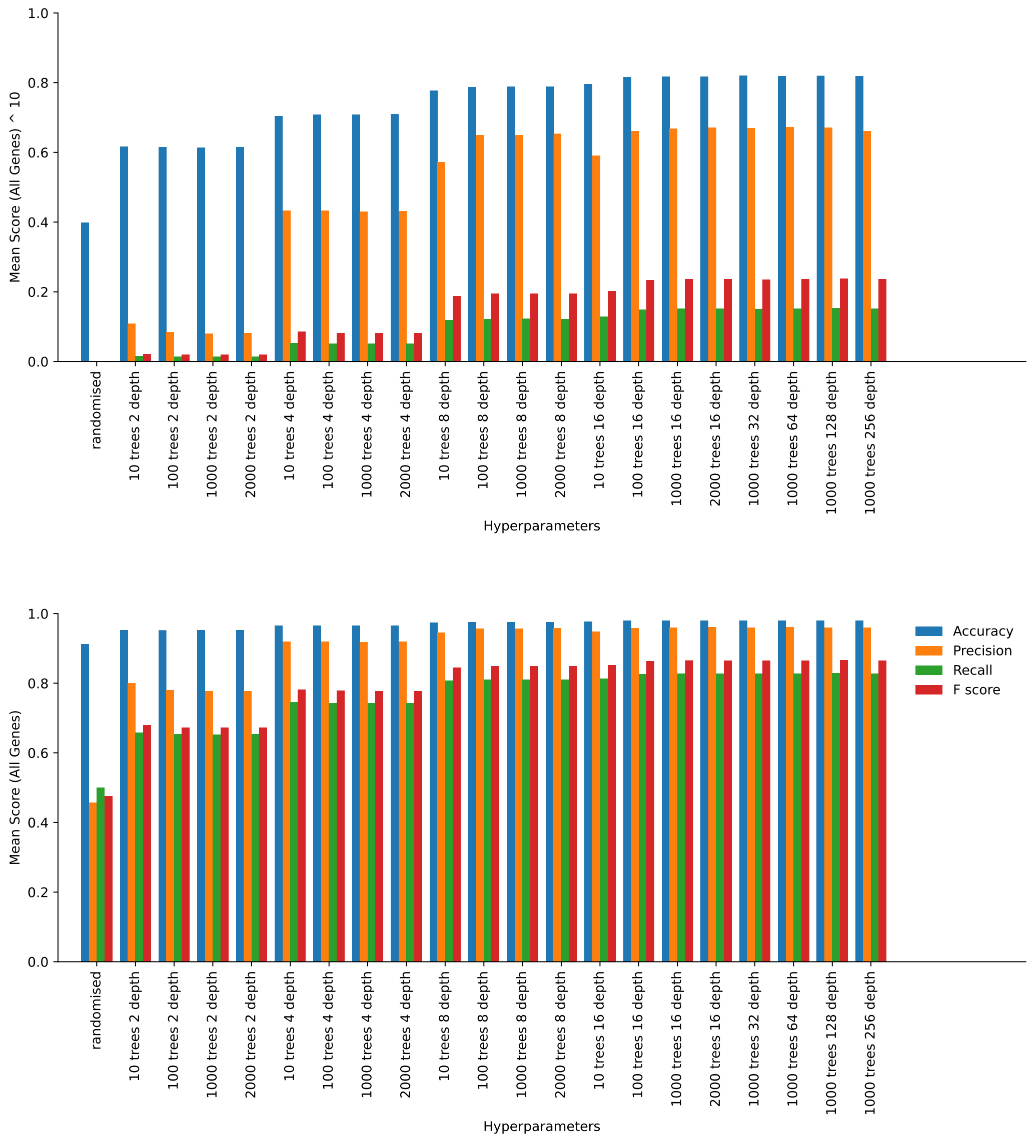

Supplementary figure 1: The average Accuracy, Precision, Recall and F-Score for all genes in random forest analyses with varying numbers of trees and maximum tree depth. The values have been transformed to the power of 10 (A) to emphasise the differences between hyperparameter sets. The randomised dataset features genes which are randomly assigned the genomes that they occupy, retaining the total number of genomes a given gene is found in, assessed using 1000 trees at a maximum depth of 16. In all cases the performance metrics relate to the predictions made in the test set of genomes (not the training set).

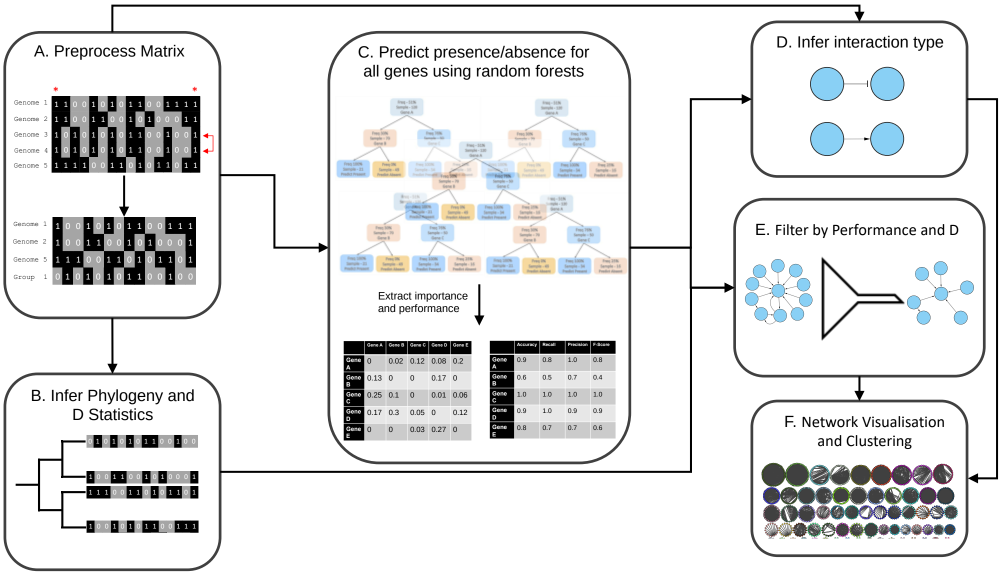

Supplementary Figure 2: An illustration of the analytical pipeline taken in this study. Arrows indicate that the output of one step serves as the input to the next.

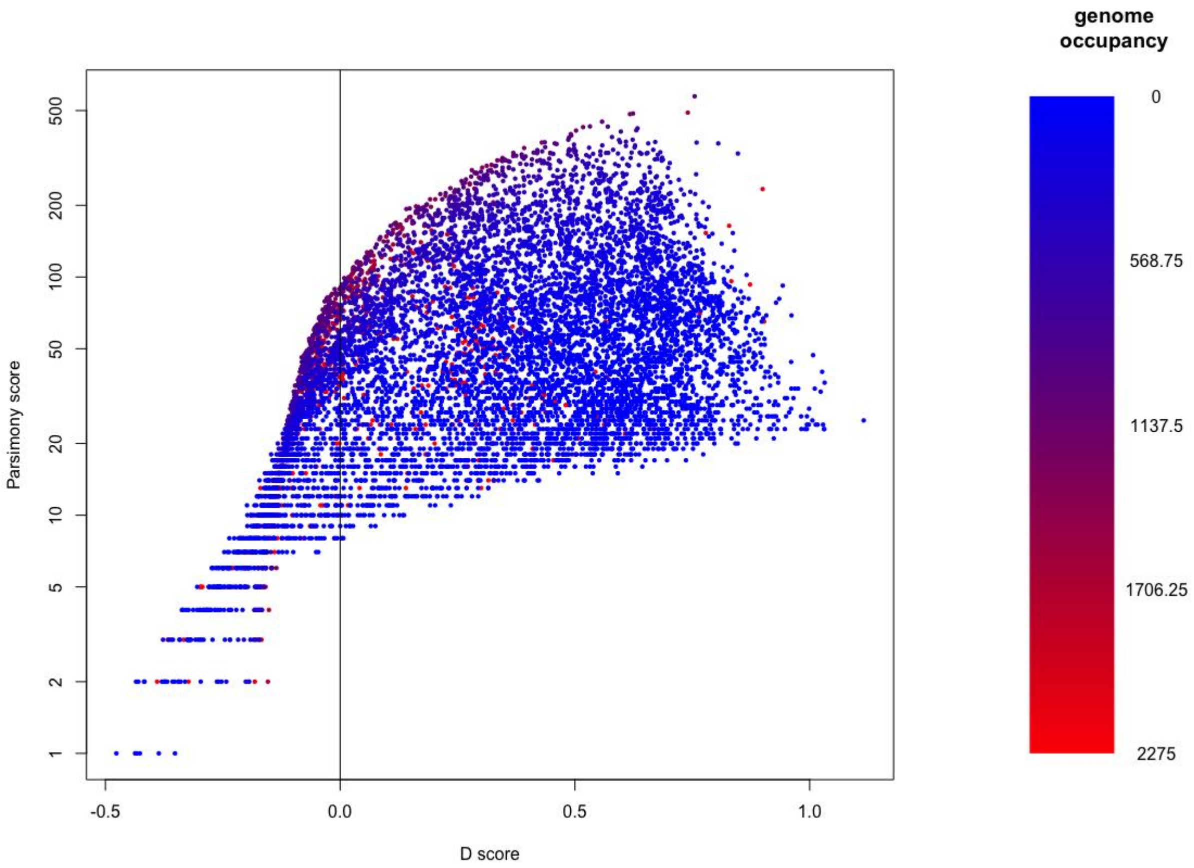

Supplementary Figure 3: The relationship between parsimony score (y axis), D score (x axis) and number of genomes in which a gene in present (colour scale). The vertical black line at D score = 0 represents the point below which genes were excluded from our results.

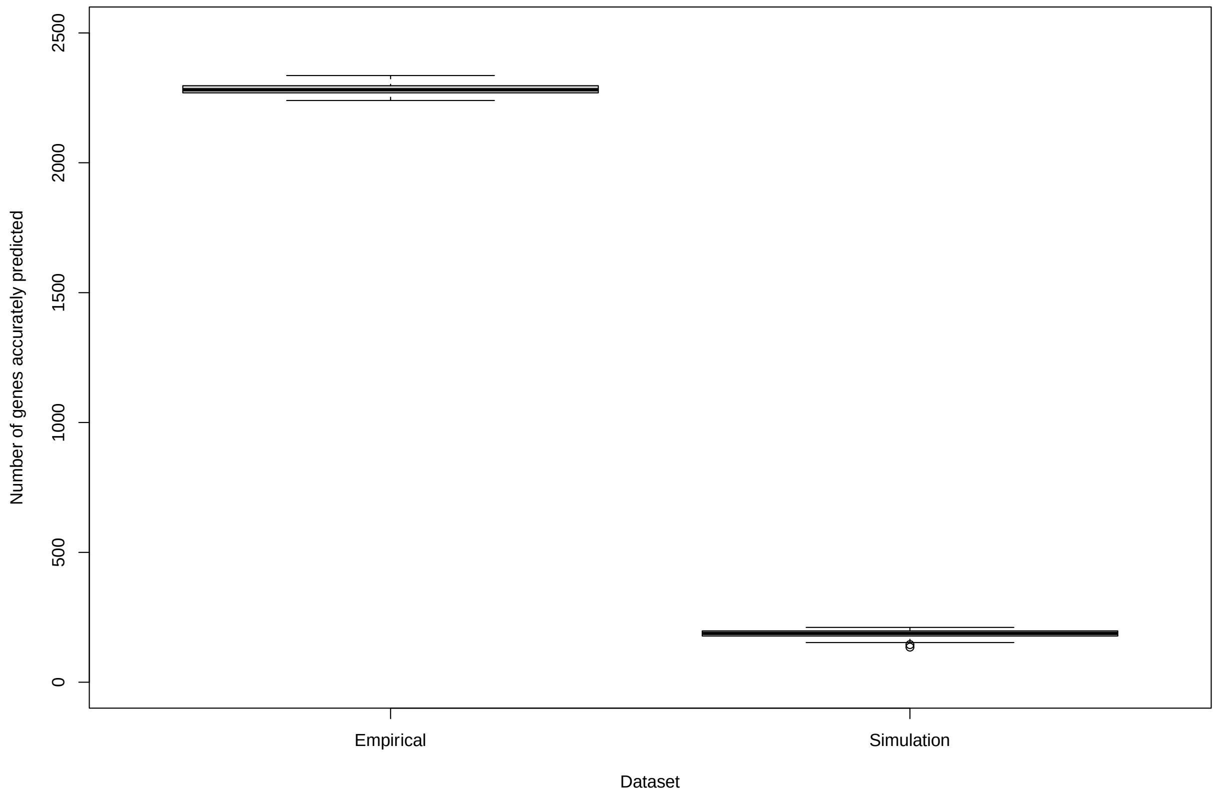

Supplementary figure 4: A comparison of the performance of random forests on the empirical dataset compared with the simulated dataset. The number of genes with a D score < 0 and accurately predicted in 100 independent analyses of the empirical dataset and separate simulated datasets are shown in a box and whiskers plot. A dark line within the boundaries of the box marks the median number of genes across each analysis. The box represents the interquartile range, and the whiskers extend to 1.5 times the interquartile range beyond the median or, if no outliers are found, the most extreme datapoint. Outliers are values outside the whisker range and are represented by circles.

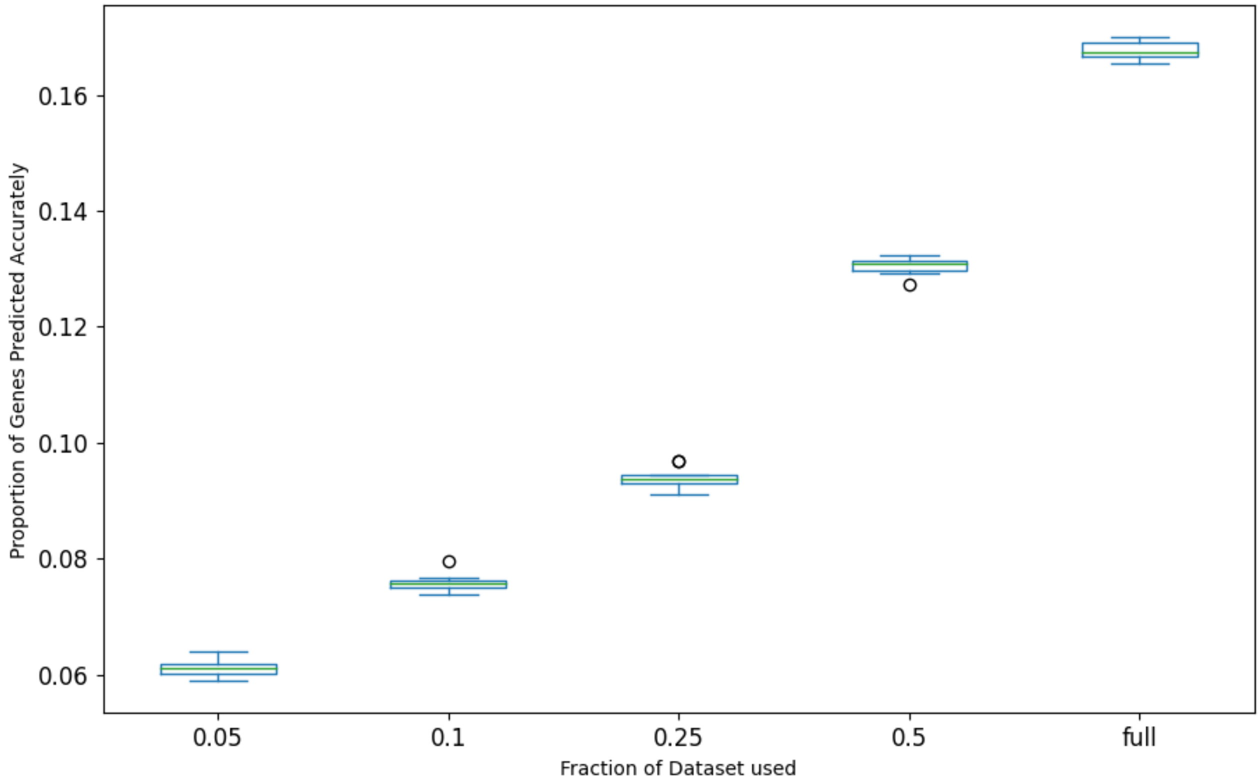

Supplementary Figure 5: The proportion of genes predicted accurately after filtering by D-score in a series of experiments where the dataset was down sampled according to the X axis. In each boxplot, the green line represents the median proportion of gene families predicted accurately across 10 repeats, the blue lines between which the box lies are the lower and upper quartiles and the outermost blue horizontal lines are minimum and maximum proportion of genes predicted accurately within 1.5 times the interquartile range of the upper or lower quartile. Values outside this range are plotted as circles.

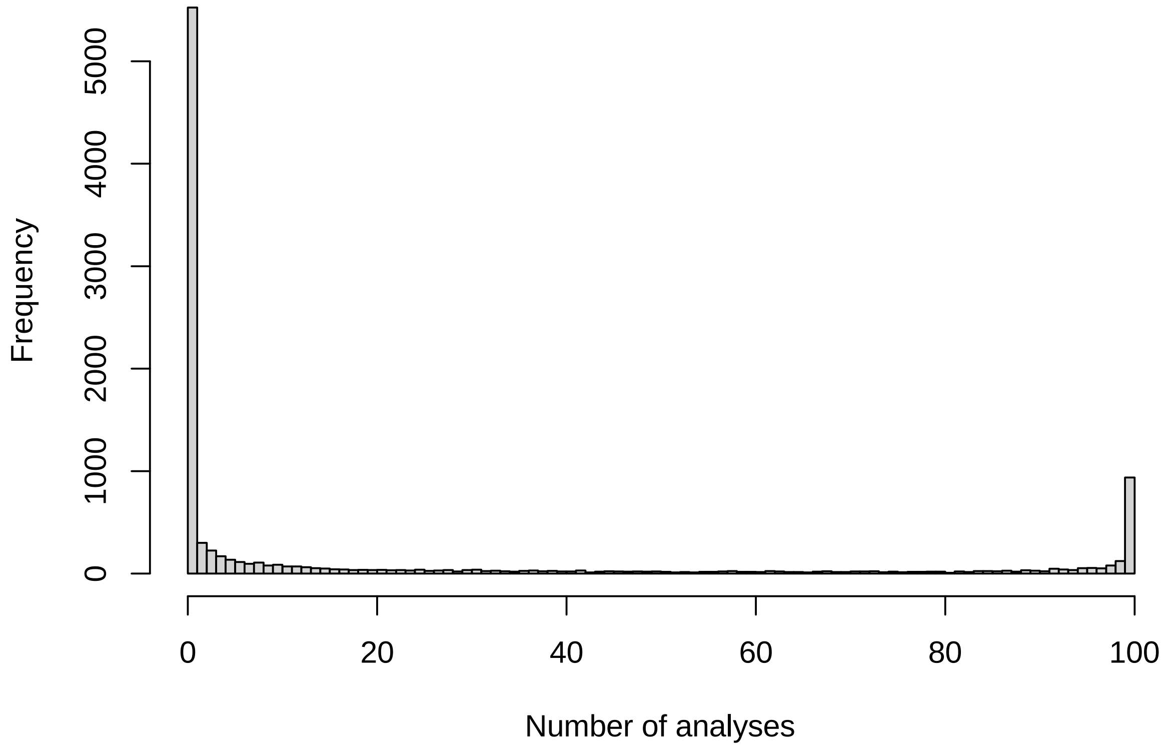

Supplementary Figure 6: Histogram showing the number of repeated analyses in which a given gene is found to be predictable providing it has a D score > 0.

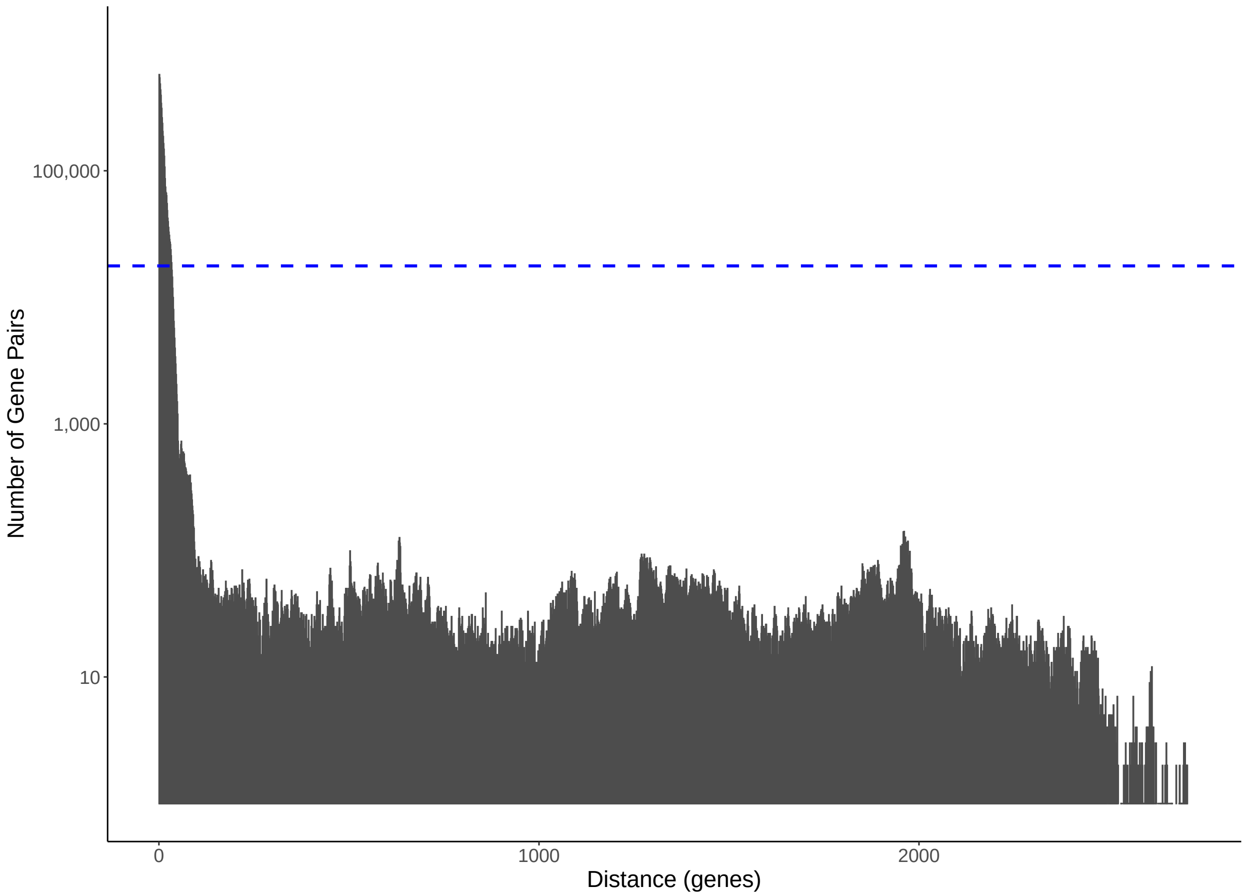

Supplementary figure 7: Most genes in associated pairs including the most highly predictable genes are closely linked. The number of associations (log transformed y axis) are plotted against physical distance in number of genes (x axis). A horizontal blue dashed line marks the number of coincident gene pairs in the same genome where each gene occupies a different genomic element (usually chromosome and plasmid).

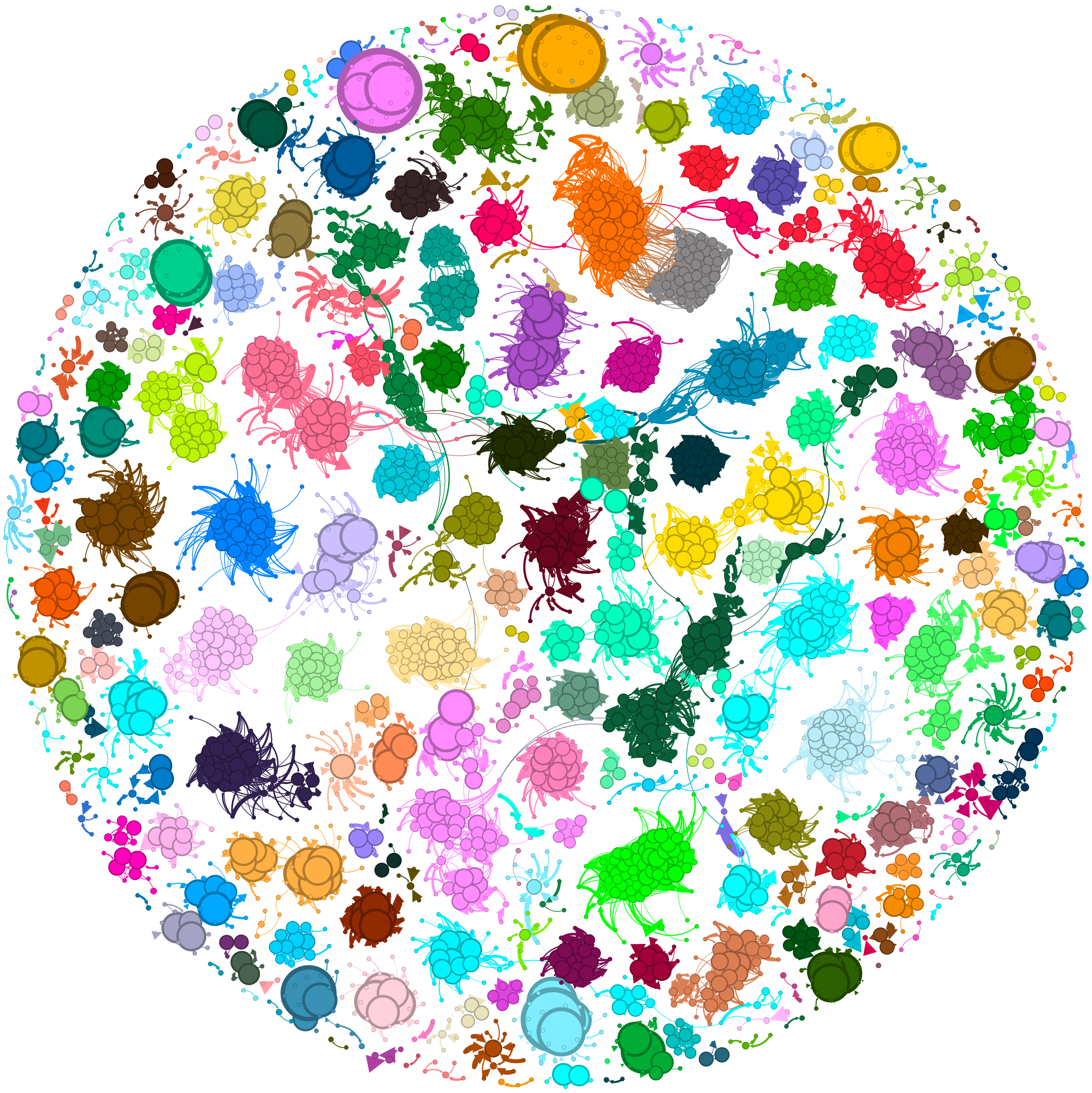

Supplementary Figure 8: Coincident relationships between accurately predicted genes and their predictors excluding predictor genes with a D score less than 0. As in Figure 1, node size is proportional to its Page Rank and edge with is proportional to GINI importance of the source node in predicting the target node. The difference between this figure and figure one is therefore only that 273 source nodes are absent here and, by extension, 34 target nodes that are predicted only by genes with D < 0.

Supplementary table 3: enriched and purified GO terms in the least predictable set of genes.

GO	Type	Enriched (e) or Purified (p)	Name	Ratio In Study	Ratio in Population	P (FDR)
GO:0019700	BP	e	organic phosphonate catabolic process	5/939	11/12840	0.049850694
GO:0008150	BP	p	biological_process	62/939	1781/12840	9.04E-10
GO:0009987	BP	p	cellular process	48/939	1420/12840	5.36E-08
GO:0044237	BP	p	cellular metabolic process	28/939	824/12840	0.000834046
GO:0008152	BP	p	metabolic process	35/939	946/12840	0.000834046
GO:0044238	BP	p	primary metabolic process	24/939	733/12840	0.000880626
GO:0071704	BP	p	organic substance metabolic process	30/939	831/12840	0.001420432
GO:0043170	BP	p	macromolecule metabolic process	17/939	581/12840	0.001477966
GO:0044260	BP	p	cellular macromolecule metabolic process	14/939	512/12840	0.001769206
GO:0006807	BP	p	nitrogen compound metabolic process	20/939	618/12840	0.003074549
GO:0090304	BP	p	nucleic acid metabolic process	11/939	431/12840	0.003074549
GO:0034641	BP	p	cellular nitrogen compound metabolic process	15/939	513/12840	0.003074549
GO:1901360	BP	p	organic cyclic compound metabolic process	15/939	505/12840	0.005100573
GO:0006725	BP	p	cellular aromatic compound metabolic process	15/939	500/12840	0.00632244
GO:0046483	BP	p	heterocycle metabolic process	15/939	492/12840	0.007646699
GO:0006139	BP	p	nucleobase-containing compound metabolic process	15/939	470/12840	0.02092484
GO:0033554	BP	p	cellular response to stress	4/939	234/12840	0.02092484
GO:0006950	BP	p	response to stress	8/939	317/12840	0.02829274
GO:0044419	BP	p	biological process involved in interspecies interaction between organisms	3/939	201/12840	0.030268514
GO:0050896	BP	p	response to stimulus	16/939	468/12840	0.036434635
GO:0051179	BP	p	localization	8/939	305/12840	4.06E-02
GO:0009058	BP	p	biosynthetic process	9/939	326/12840	4.06E-02
GO:1901576	BP	p	organic substance biosynthetic process	9/939	325/12840	0.040578801
GO:0110165	CC	p	cellular anatomical entity	45/939	1254/12840	1.02E-06
GO:0005575	CC	p	cellular_component	52/939	1371/12840	1.02E-06
GO:0016020	CC	p	membrane	21/939	634/12840	0.001070468
GO:0071944	CC	p	cell periphery	21/939	616/12840	0.001792367
GO:0030313	CC	p	cell envelope	4/939	221/12840	0.009825618
GO:0031975	CC	p	envelope	4/939	221/12840	0.009825618
GO:0044217	CC	p	other organism part	1/939	115/12840	0.040512529
GO:0033643	CC	p	host cell part	1/939	115/12840	0.040512529
GO:0043657	CC	p	host cell	1/939	115/12840	0.040512529
GO:0005886	CC	p	plasma membrane	18/939	456/12840	0.040512529
GO:0018995	CC	p	host cellular component	1/939	116/12840	4.05E-02
GO:0009279	CC	p	cell outer membrane	3/939	161/12840	0.040512529
GO:0019867	CC	p	outer membrane	3/939	163/12840	0.040512529
GO:0003674	MF	p	molecular_function	41/939	1253/12840	3.34E-07
GO:0003824	MF	p	catalytic activity	24/939	771/12840	0.000234463
GO:0016787	MF	p	hydrolase activity	3/939	285/12840	0.000465117
GO:0005488	MF	p	binding	20/939	586/12840	0.015630625

Supplementary table 4: The names of genes inferred by Panaroo, their labels in the main text and the node they correspond to (figure 2 main text)

Gene (group)	Name in main text	Full Panaroo annotation
A	*farR*	farR
A	*hpcG*	hpcG
A	*ttuB*	ttuB_1~~~ttuB_2~~~ttuB
A	*hpcB*	hpcB~~~hpcB_1~~~hpcB_2
A	*hpcE*	hpcE~~~hpcE_1~~~hpcE_2
A	*hpcD*	hpcD
A	*hpcH*	hpcH_2~~~hpcH~~~hpcH_1
A	*iolA*	iolA~~~xylG_2~~~betB_1~~~xylG_1~~~tgnC~~~aldB_1~~~aldB_2~~~betB_2
A	*hpaB*	hpaB_2~~~hpaB~~~hpaB_1
B	*hpaC*	hpaC
C	*rhaR*	rhaR_2~~~rhaR_4~~~rhaR_3~~~rhaR_5
D	group_39613	group_39613
E	*pac*	pac_1~~~pac_3~~~pac~~~pac_2
F	*symE*	symE~~~symE_3~~~symE_8~~~symE_1~~~symE_2~~~symE_4
G	group_13180	group_13180
H	group_19718	group_19718
I	*hsdM*	hsdM
I	*mrr*	Mrr

Supplementary table 5: The names of genes inferred by Panaroo and how we refer to them in the main text

Gene (group)	Name in text	Name given by Panaroo
J	*lgoT*	lgoT_1~~~lgoT~~~lgoT_2
K	*mdtM*	mdtM~~~mdtM_2~~~mdtM_1~~~mdtL_1
L	*nhaK*	nhaK_2~~~nhaK_1
L	*siaP*	group_24769
L	*siaT*	siaT~~~siaT_2
M	*dctM:siaM*	dctM~~~dctM_2~~~dctM_1~~~siaM_2~~~siaM_1
